# Supplementary material for: Patterns of Intron Gain and Loss in Fungi
Source: PLoS Biol. 2004 Nov 30;2(12):e422. doi: 10.1371/journal.pbio.0020422 (PMC532390; doi:10.1371/journal.pbio.0020422)
Supplement: Table S1 — Also available at http://genes.mit.edu/NielsenEtAl/. (4.3 MB ZIP). [file pbio.0020422.st001.zip › NielsenEtAl/html/1310.html]

AN4830.1.NCU09090.1.MG02725.1.FG08803.1


```
 CLUSTAL W (1.82) Multiple Sequence Alignments - Introns Inserted


Sequence 1: MG02725.1	439 aa
Sequence 2: FG08803.1	365 aa
Sequence 3: NCU09090.1	440 aa
Sequence 4: AN4830.1	378 aa
Alignment Length: 464 aa
Number Identitical Residues: 169 aa
Alignment Score (without introns) 8161


MG02725.1 	MSNVPQPPKPAGAEPSAAAEEDHYFSSNPPPRGLSEHVALASAFIEQHAAANRRVVLVTS
NCU09090.1	--MMSQIPGETPPPNLATLAEDHYFSANPPPKDLEKHVKQAKDFIDFHAKEGRRLVLITS
FG08803.1 	------MATPANTTAEALASEEAYFSQNPPPRNLAAHTALADGFISSHAAAGRRVVLVTS
AN4830.1  	--------------MSPADSESAYFNNYPPPKALSKHESLARSFIEYHVESSRRVVLVTS
          	                .   *. **.  ***: *  *   *  **. *.  .**:**:**

MG02725.1 	GGTTVPLE~RQTVRFIDNFSAGTRGA~TSAEYFLEAG~YAVIFLHRQFSLLPYSRHYSHT
NCU09090.1	GGTTVPLE1KQTVRFIDNFSAGTRGA0TSAEYFLEAG~YAVLFLHRQFSLLPFSRHYSHA
FG08803.1 	GGTTVPLE~KQTVRYIDNFSAGTRGA~TSAEYFLEAG~YAVIFLHRQFSLLPYSRHYSHA
AN4830.1  	GGTTVPLE~NQTVRFIDNFSAGTRGA~TSAEYFLEQG2------HSTNCFLDFM------
          	******** .****:*********** ******** *       *   .:* :       

MG02725.1 	PTDCFLDFLHEENGAVVAEPGR-AEKMLGVLRKYRAAKQGNMLLMLPFVTITDYLYELRA
NCU09090.1	-KDCFLDFLREGPDGSVVARDKDASKMLQVLRKYHDARDNNMLLAIPFLSISDYLHELRS
FG08803.1 	-TDCFLDFLTEDADDRIAVRGEVQDKMREVLRKYRKARDNNMLLTLPFVTIVDYLHELRE
AN4830.1  	-DEAFPSDVSRSDHGPIVVRKEYQDEMRDVLRKYRYAKQNNLLLLLPFTTVSEYLFELRM
          	  :.* . : .     :.   .  .:*  *****: *::.*:** :** :: :**.*** 

MG02725.1 	VAQLMRPLGPRGLLYLAAAVSDFFVPPERMAEHKIQSTTAVERMKHSSSSTERKPQAQAA
NCU09090.1	IAQLMKPLGPNALLYLAAAVSDFFVPPDRMSEHKIQSTDAADLKKKLSSSTATSSAAPSA
FG08803.1 	IARLMRPLGPSGLLYLAAAVSDFFVPPDRMAEHKIQSTNAVE------------------
AN4830.1  	LAKCMNPLGPNALFYLAAAVSDFFIPRDRMAEHKIQSSEIPKEFQ---------------
          	:*: *.**** .*:**********:* :**:******:   .  .               

MG02725.1 	PPLGALPIVPIPPGEQPLQQPTTLSAEELNTAVAKEQAAADDETFDNFDSSPAVPRSKRL
NCU09090.1	NP--------------SARATPASGGSVGSRNGSGGTGVVDEETFDNFDSSPTVPRSKRL
FG08803.1 	-----------------------------------QKRTEEEETFDNFDSSPSVPRSKRL
AN4830.1  	--------------------------------GNDEAVGADDLYTGGFEQKQ--ESSKKL
          	                                        ::   ..*:..     **:*

MG02725.1 	IVDLDPVPKFLKNLVDGWAPQG-MIVSFKLETDPAILVHKARYSLDRYQHHLVIGNLL~S
NCU09090.1	VVDLDPVPKFLKNLVEGWSPEG-MIVSFKLETDPSILTLKAKYSLNRYQHHLVIGNLL2S
FG08803.1 	IVDLDPVPKFLKNLVDGWAPEG-MIVSFKLETDPALLVRKARYSLDRYQHHLVIGNLL~S
AN4830.1  	VINLDPVPKFLHQLVDGWSPEGSMIVSFKLETDPNLLVYKAQTALQRYAHHLVIGNLL~S
          	:::********::**:**:*:*:*********** :*. **: :*:** ********* *

MG02725.1 	TRKWEVVFVAPGA-ADRWIRVPTGRRRGSVSGREDMVG----------AAEAKMRVAEMP
NCU09090.1	TRKWEVVFVAPNR-KDRWIRVPMHRRVGGSSSTSTSSGNPAAAVT---TTTTTTTDKDEP
FG08803.1 	TRKWEVVFVSPRR-DDRWVRVP----------REGGWG----------EAE------ERP
AN4830.1  	TRKWEVVFVTPNPPYERWIRVPKSRRSKSISGVEDQVGKAEAAKRSSGDQTLAAPVGEEP
          	*********:*  . :**:***      . :. .   *.. ::  ::.         : *

MG02725.1 	LDPASLPEGEPE--MEIESLIIPAVDELHTRHIKSIAP----------
NCU09090.1	LDPKSLPEGEPE--IEIESLIIPAVKDLHSEYIDTFERERRGRSGTPA
FG08803.1 	LVAEELPAQDPD--LEIESLIIPAVQELHGEHIKGQSKK---------
AN4830.1  	SKEEKDGEGTSREGTEIESLIIPELVKLHSEMIEKFKR----------
          	    .     . .. ******** : .** . *.
```
